# Detection of rare prostate cancer cells in human urine offers prospect of non-invasive diagnosis

**DOI:** 10.1038/s41598-022-21656-9

**Published:** 2022-11-02

**Authors:** Nima Sayyadi, Irene Justiniano, Yan Wang, Xianlin Zheng, Wei Zhang, Lianmei Jiang, Dmitry M. Polikarpov, Robert D. Willows, David Gillatt, Douglas Campbell, Bradley J. Walsh, Jingli Yuan, Yiqing Lu, Nicolle H. Packer, Yuling Wang, James A. Piper

**Affiliations:** 1 School of Natural Sciences, Macquarie University, Sydney, Australia; 2 ARC Centre of Excellence for Nanoscale Biophotonics (CNBP), Macquarie University, Sydney, Australia; 3 Minomic International Ltd, Macquarie Park, Sydney, Australia; 4 Department of Physics and Astronomy, Macquarie University, Sydney, Australia; 5 Faculty of Medicine and Health Sciences, Macquarie University, Sydney, Australia; 6 State Key Laboratory of Fine Chemicals, School of Chemistry, Dalian University of Technology, Dalian, China; 7 School of Engineering, Macquarie University, Sydney, Australia

**Keywords:** Cancer, Immunology, Biomarkers

## Abstract

Two molecular cytology approaches, (i) time-gated immunoluminescence assay (TGiA) and (ii) Raman-active immunolabeling assay (RiA), have been developed to detect prostate cancer (PCa) cells in urine from five prostate cancer patients. For TGiA, PCa cells stained by a biocompatible europium chelate antibody-conjugated probe were quantitated by automated time-gated microscopy (OSAM). For RiA, PCa cells labeled by antibody-conjugated Raman probe were detected by Raman spectrometer. TGiA and RiA were first optimized by the detection of PCa cultured cells (DU145) spiked into control urine, with TGiA-OSAM showing single-cell PCa detection sensitivity, while RiA had a limit of detection of 4–10 cells/mL. Blinded analysis of each patient urine sample, using MIL-38 antibody specific for PCa cells, was performed using both assays in parallel with control urine. Both assays detected very low abundance PCa cells in patient urine (3–20 PCa cells per mL by TGiA, 4–13 cells/mL by RiA). The normalized mean of the detected PCa cells per 1 ml of urine was plotted against the clinical data including prostate specific antigen (PSA) level and Clinical Risk Assessment for each patient. Both cell detection assays showed correlation with PSA in the high risk patients but aligned with the Clinical Assessment rather than with PSA levels of the low/intermediate risk patients. Despite the limited available urine samples of PCa patients, the data presented in this proof-of-principle work is promising for the development of highly sensitive diagnostic urine tests for PCa.

## Introduction

Prostate cancer (PCa) is the most commonly diagnosed malignancy and the second leading cause of cancer deaths in the western male population^1^. Annual prostate cancer diagnoses worldwide are projected to rise from 1.4 million in 2020 to over 2 million in the next 20 years^2^.

The gold standard for the diagnosis of PCa is a prostate biopsy. The decision to perform a prostate biopsy generally relies on the digital (finger) rectal examination (DRE) of the prostate gland and the prostate-specific antigen (PSA) levels in the blood^3^. PSA level is a simple-to-perform blood test, so for the last 20 years it has become widespread and has helped to detect PCa^4^. However, its low specificity (33%)^5^ has also been increasingly recognized as a major drawback^6^. As a result, a high PSA level has led to many unnecessary biopsies and the overtreatment of low-risk PCa patients^7–9^. Follow-up prostate biopsy is invasive and can be painful and the side effects are sometimes serious^10^. More than half of these biopsies are negative for PCa due to the fact that the PSA biomarker in the serum can be elevated for reasons other than PCa^11–13^. Therefore, there is a clinical need for sensitive, and non-invasive diagnostic tests that can better discriminate between the presence and absence of prostate cancer.

The low sensitivity of serum PSA test as a major factor in deciding to perform a prostate biopsy has resulted in a great efforts to be carried out worldwide for more sensitive PCa biomarkers. Radiomics imaging approaches such as multi-parametric magnetic resonance of the prostate (mpMRI) and prostate-specific membrane antigen positron emission tomography (PSMA-PET) and new ultrasound scans are also in use for PCa screening and diagnosis^9^. As a result, crucial changes in the 2020 European Association of Urology (EAU) guidelines were established, promoting mpMRI to being recommended initially for every patient with suspicion of PCa (elevated PSA/abnormal DRE) before performing the biopsy^14^. Bourdoumis et al.^15^ reported a prostate-specific, non-protein coding RNA (PCA3) that is significantly overexpressed in PCa. PCA3 has the potential, in combination with a PSA test and a DRE examination, to improve the decision about whether to perform a biopsy or not. The SelectMDx test is another urine-based mRNA based biomarker test which measures two cancer-related genetic biomarkers (HOXC6 & DLX1)^16^. The SelectMDx test in combination with clinical risk factors has high sensitivity for the detection of high-grade PCa and can be used to select patients at risk for high-grade PCa for further diagnostics. Tomlins et al.^17,18^ has identified repeated gene fusions of TMPRSS2 to ERG in PCa tissues with outlier expression that can detect PCa non-invasively in urine. TMPRSS2:ERG is a highly specific tissue biomarker for prostate cancer and combination with PCA3 can improve on the risk assessment. The ExoDx Prostate test is a urine exosome gene expression assay that does not require pre-collection digital rectal exam (DRE). It was shown in clinical setting to improve the ability to identify clinically significant PCa disease and reduce biopsies if the test is negative^19^. However, the sensitivity of available PCa biomarkers is still not sufficient, thus more sensitive biomarkers for PCa presence, progression and response to intervention are needed to avoid unnecessary treatment.

Although the prevalence of intact PCa cells in urine sediment has not been systematically investigated, it is known that PCa cells are shed into the urine through the urethra, and thus provide a non-invasive means to detect PCa in an easily obtainable body fluid^20,21^. Detecting PCa cells in the urine using conventional molecular cytology tests such as immunofluorescence assay (IFA) has unacceptably low sensitivity, although the specificity is typically high^22,23^. The lack of sensitivity is mostly due to the low numbers of PCa cells present in the urine samples, further losses in cell numbers in processing the urine sample, plus the difficulty in differentiating fluorescently labelled malignant PCa cells from the auto-fluorescent background due to other cells and debris seen on the stained urine cytology slide. These limitations of IFA for urinary PCa cell detection were reported by Campbell et al.^23^ in which 40% of cytoslides from patients’ urine samples were not analysed either due to the low numbers of cancer cells or high auto-fluorescence background. Consequently, detecting prostate cancer cells via urine cytology as diagnostic approach has been largely abandoned^21^.

**Figure 1:**
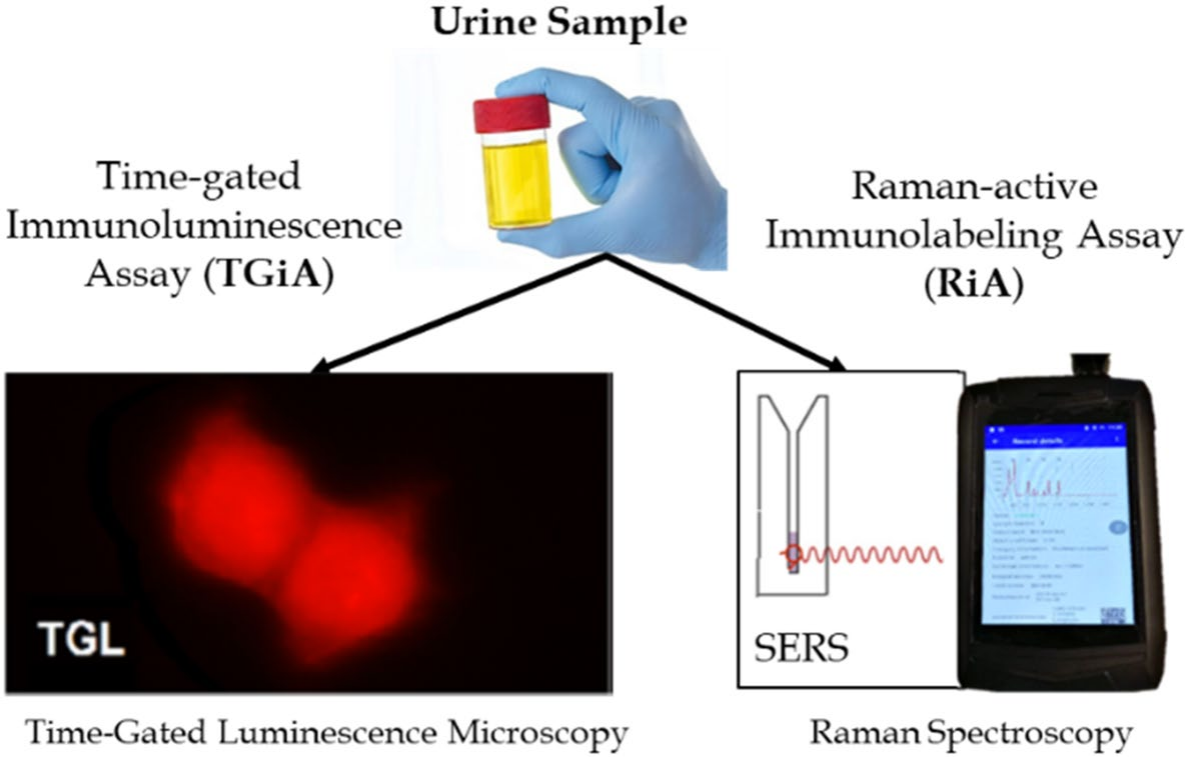
TGiA and RiA assays for immunodetection of PCa cells in (male) patient’s urine.

Here, we report two different molecular cytology approaches for the detection of PCa cells in human urine samples (i) a time-gated immunoluminescence assay (TGiA) using a luminescent europium probe to detect and quantitate rare PCa cells and (ii) a Raman-active immunolabeling assay (RiA) to sensitively and rapidly detect PCa cells in the urine of prostate cancer patients (Fig. 1) using the commercially available prostate cancer specific antibody (MIL-38 antibody). The MIL-38 antibody target is glypican-1 (GPC-1), a proteoglycan that is on the cell surface of prostate cancer cells isolated from patient urine^24–26^.

## Materials and methods

### Reagents

Polycarbonate membrane filters (13 mm, 8 µm pore size, TETP01300, Millipore) were purchased from Merck Australia. Pop-Top filter membrane holder (Whatman 420100-13mm), Bovine Serum Albumins (BSA) (A2058), Trioctylphosphine oxide (223301), 4′,6-Diamidino-2-phenylindole dihydrochloride hydrate (DAPI) (D9642), Europium (III) chloride hexahydrate (203254), Tween-20 (P1379), Ethanolamine (E9508), Glycerol (G5516), 5,5′-Dithiobis (2-nitrobenzoic acid) [DTNB] (D8130), 4-Mercaptobenzoic acid (MBA) and Gold (III) chloride trihydrate (HAuCl_4_) were purchased from Sigma-Aldrich Australia. 2,3,5,6-Tetrafluoro-4-mercaptobenzonic acid (TFMBA) was purchased from Tokyo Chemical Industry. Secondary Antibody (anti-mouse IgG Alexa-Fluor 488), 3,3′-dithiobis (sulfosuccinimidyl propionate (DTSSP) (803200) and Saccomanno fixative (76161) were purchased from Thermo-fisher Scientific. The Sephadex column (PD MiniTrap G-25) (28-9180-07) was purchased from GE Healthcare Life Sciences, Australia. Human PCa cell line DU145, bladder cancer cell line C3 and mouse monoclonal IgG (MIL-38) were provided by Minomic International.

### Preparation of europium chelate conjugated to secondary antibody

We have previously reported conjugation of a novel europium chelate (BHHBTEGSB-Eu^3+^) to anti-mouse IgG antibody^27^. Briefly, for the conjugation reaction, 100 μg anti-mouse IgG antibody was exchanged into NaHCO_3_ buffer (100 mM, pH 8.5) and then mixed with 15 molar excess of the BHHBTEGSB ligand. After incubation for 1 h at 37 °C, 50μL of ethanolamine solution were added to stop the reaction, then the reaction mixture passed through a Sephadex column (PD MiniTrap) using Tris Buffered Saline (TBS) (20 mM, pH 7.5, 150 mM NaCl) containing 5% (v/v) glycerol as an eluent to purify the conjugated Ab from excess of europium ligand. The fractions corresponding to labelled conjugates were collected according to their absorbance detection measured by an Eppendorf BioPhotometer (at 280 and at 320 nm).

### Time-gated immunoluminescence assay (TGiA)

The following TGiA labelling assy was first optimized for spiked PCa culture cells (DU145) in control urine (Supplementary Information) and used for the patients samples. The fixed cells captured from the patient urine on the filter membrane were washed with TBS (3 times, 1 mL) and then incubated with blocking solution [500 µL of 1% BSA (w/v) in TBS] for 30 min, followed by washing the filter with TBS (3 times, 1 mL). The filter was gently transferred from the filter assembly holder to the microscope slides and incubated with primary antibody MIL-38 (0.5 mg/mL) in blocking solution for 2 h at RT or overnight at 4 °C. The filter was gently washed with TBS (3 times) and then anti-mouse IgG conjugated europium chelate (0.5 mg/mL) was added in blocking solution and incubated for 2 h at room temperature. The filter was washed with TBS containing 0.1% v/v Tween-20.

Mounting solution [5 μL DAPI (2 μg/mL in TBS with 70(v/v) % glycerol) and 5 μL of europium chloride (20 mM) in 15 μL of the fluorescence enhancing buffer (FEB)] was added to enhance intensity of luminescence emission of europium probe in the time-gated luminescence (TGL) imaging and incubated for 15 min before being covered with a coverslip and inspected by TGL-OSAM microscope. FEB buffer consists of Trioctylphosphine oxide (TOPO, 0.5 mM), Tween-20 (1%, v/v) in acetate buffer (100 mM, pH 5.5)^26^.

### Preparation of SERS nanotags for Raman-active immunolabeling assay (RiA)

Gold nanoparticles (AuNPs) were synthesized by the reduction of HAuCl_4_ by citrate solution as reported by Frens^28^. SERS nanotags were prepared as our previous report. Briefly, 20 µL of 1 mM Raman reporter 5,5′-Dithiobis (2-nitrobenzoic acid) (DTNB) was added into the AuNPs and incubated at room temperature overnight with shaking (60 rpm) to form AuNPs-DTNB. The mixture was then centrifuged at 7000 rpm (4602 RCF) for 5 min to remove residual reactants and re-suspended in 1 mL Milli-Q water. Antibody with DTSSP linker was prepared by mixing 10 µl 1 mg/ml of DTSSP (5 mM sodium citrate solution pH = 5.3) and 20 µl of 0.15 mg/ml MIL-38 at 300 rpm and shaking in RT for 30 min. SERS nanotags was thus prepared by mixing MIL-38-DTSSP and AuNPs-DTNB at 300 rpm shaking in RT for 30 min and at 4℃ overnight. The mixture was centrifuged (300 rpm) to remove free antibodies and then mixed with BSA (500 µl, 0.05% (w/v) in 0.1 mM PBS to block non-specific binding at RT for 1 h under 350 rpm shaking. After removing the free BSA, AuNPs-DTNB -MIL-38 antibody (SERS nanotags) were ready to use in RiA.

### Raman-active immunolabeling assay (RiA)

SERS nanotags (30 µL) were mixed with re-suspended cells in PBS (1 mL) of cells collected from urine, and were incubated at 37 °C for 45 min under gentle shaking (300 rpm). The solution of the SERS nanotags and cells was then centrifuged at 500 g for 5 min to remove excess SERS nanotags, as at 500 g the unbound SERS nanotags are a stable colloidal suspension while the SERS nanotags bound to the target cells pellet in the centrifuge. Finally, 60 µl PBS was added to the tagged cell pellets and the solution was analysed in a quartz cuvette with a Raman spectrometer at 785 nm excitation (Snowy Range-IM52, with the Raman shift range from 800 to 1600 cm^−1^).

### Optimization of TGiA and RiA assay using spiked PCa cells (DU145) in control urine

PCa cells were spiked into the urine of healthy male volunteers for the development and optimization of the TGiA and RiA assays before carrying out the tests in PCa patients’ urine. From these experiments the fixation and filtration of urine samples, specificity, and sensitivity and automated microscopy of TGiA assay, and the Raman-active Immunolabeling Assay specificity and sensitivity and LOD of the RiA assay were determined (Supplementary Information (SI)).

### Collection and processing of human urine samples

Fresh urine specimens (40–90 mL) from 5 prostate cancer patients prior to biopsy were collected without prior Digital Rectal Examination (DRE) (Ethics 5201500707). Additional control urine samples were similarly collected from five healthy male volunteers.

Urine samples of patients were filtered through a polycarbonate membrane filter (8 µm pore size) as shown in Fig. S1. Different urine volumes were selected for analysis based on the total numbers of urinary cells per mL after initial analysis of patient urine sample by fixation, filtration, DAPI labelling and manual cell count by epifluorescence microscopy. The urine sample volumes were adjusted to bring the total number of urinary cells per mL into 100–200 total cells.

The blood PSA level, Gleason score and clinical stage of the prostate cancer disease, which are widely accepted indicators of PCa severity and aggressiveness^29,30^, were recorded and interpreted by our expert clinician author (D. Gillatt) for risk stratification (Table 1). We note that prior DRE in collection of urine samples has previously been identified as a significant source of variability in analytical results^21^. Taking account of this and patient comfort led us to eliminate DRE as part of the collection protocol and sample source was blinded for all experiments.

**Table 1:** Clinical data of patients including PSA, Gleason score, clinical stage and clinician based judgement of risk.

	PSA (ng/mL)	Gleason score	Clinical Stage	Risk	TGiA assay					RiA assay					Adjusted* urine volume (mL) tested for each replicate
					PCa cells detected			Mean PCa cells normalized per 1 mL of urine*	SD	PCa cells detected			Mean PCa cell normalized per 1 mL of urine**	SD	
					Replicates					Replicates					
Patient 1	13	Not available	cT1c	High	14	8	11	11	3	9	5	13	9	4	1
Patient 2	7.9	9 (4 + 5)	cT3M1	High	3	11	0	7	6	4	13	0	9	7	1
Patient 3	8.7	7 (3 + 4)	cT1c	Intermediate	20	12	7	4.3	7	12	13	5	3.3	4	3
Patient 4	14	7 (3 + 4)	cT1c	Intermediate low	0	0	0	0	0	0	0	0	0	0	2
Patient 5	0.24	9 (5 + 4)	cT1a	Low	0	0	0	0	0	0	0	0	0	0	1

The number of PCa cells detected by TGiA and RiA assays of three replicates of patients’ urine samples. OSAM microscopy was used to count the number of cells labelled with the Eu-Probe, and Raman spectroscopic signals of the RiA were approximated based on linear equation obtained from the spiked cells in urine (Fig. S8B). *The adjusted volume of patients’ urine tested for each replicate are shown in the last right column. *The mean PCa cells detected by TGiA and RiA were normalised to cells/mL.

### Instrumentation

Transmission electron microscopy (TEM) images were obtained using a JEOL-2100 system and UV–visible absorption spectra were measured with a NanoDrop 2000 UV (Thermo Scientific) spectrometer. SERS spectra were recorded with portable Raman microscope (IM-52 Snowy Range) under 785 nm excitation and a 1-s integration time at a laser power of 20 mW. Centrifuge (Eppendorf 5424R), Ultrasonicator (Unisonics), ELMI Intelli-Mixer RM-2 shaker, 37ºC shaker, ThermoMixer C were used in the preparation of SERS nanotags.

### Ethics approval and consent to participate

All experimental protocols were approved by a Macquarie University Ethics Committee (5201500707). Informed consent was obtained from all subjects. All Methods were carried out in accordance with relevant guidelines and regulations. All experimental protocols were approved by a named institutional committee.

## Results and discussion

### Selective GPC-1 MIL-38 antibody

The specificity of an immunodetection assay relies primarily on antibody performance. MIL-38, a mouse monoclonal antibody, is highly specific for the GPC-1 (Glypican) antigen expressed on the membrane of prostate cancer tissue and on PCa cells found in urine^24–26^. It is known that MIL-38 binds to the antigen glypican-1(GPC-1), which is also associated with a wide range of other tumours including prostate, bladder, pancreatic and breast carcinomas. Bladder cancer cells potentially are the only other positive MIL-38 cells that also exist in urine but it is not intended that the described tests will be done in isolation from other diagnostic investigations which will differentiate prostate from bladder cancer^26^.

### Cell capture from urine for PCa detection assays

Conventional IFA uses cytospin centrifugation for capture of cells when a large number of cells are available. For example in bladder cancer, typically thousands of tumour cells are released into the urine and so can be collected by the traditional cytospin technique^31^. In the case of PCa, the number of malignant cancer cells in urine is very low and as a result the conventional cytology assay is not sensitive enough^20,21^. Fujita et al.^31^, have shown with PCa lymph node cultured cells (LNCaP) spiked into urine, that the cytospin was only useful for over 1000 cells per mL.

The use of filtration (2, 5 and 8 µm pore size) has been previously reported as an efficient approach capable of single cell capture efficiency (Nickens et al.^32^). We have adopted the use of this filtration method (8 µm pore size) for efficient capture of urinary cells, including rare PCa cells, for the TGiA microscopic detection assay (Supplementary Fig. S1). An average capture efficiency of three replicates for 100 spiked cells was 91% and 90% from PBS and urine, respectively. Similar figures of 90% and 88% efficiency were observed when 50 spiked cells were recovered from PBS and urine and from urine samples with 10 spiked cells, around 83% and 80% of cells were recovered from PBS and urine, respectively (Fig. S1B). These results are in accordance with the previous report^32^.

### Time-gated immunoluminescence assay (TGiA)

The TGiA approach uses time-gated luminescence (TGL) microscopic techniques, to address the problem of the low sensitivity of IFA cytology in urinary PCa cell detection due to the high auto-fluorescence cell background. We conjugated a biocompatible europium chelate^27^ to a secondary anti-mouse antibody that binds MIL-38. Lanthanide chelates such as europium have unique emission characteristics, including long excited-state lifetimes, sharply spiked emission spectra and large Stokes shifts. These TGL characteristics are advantageous for discriminating against the short-lived background of cellular auto-fluorescence^33,34^. In a previous study^27^, we compared the highly sensitive immunoluminescence assay (TGiA) of culture-derived prostate cancer cells (DU145) with bladder cancer C3 cells that have a low expression of GPC-1 as a negative control^24,29^, using TGL detection.

For the work described here, TGiA was further optimized by the selective immunoluminescence labelling of DU145 PCa cells spiked into healthy urines before testing on PCa patients’ urine, as it is known that various other cells are in urine sediment, including erythrocytes, red blood cell casts, leukocyte and neutrophils, white blood cell casts and other cells of epithelial origin including renal, transitional or urothelial, and squamous^35^ that may interfere with the assay of PCa cells.

The Eu-Probe was shown to selectively labelled the PCa cells compared to other urinary cells (Supplementary Fig. S2) and the TGiA staining was about 10 times higher intensity than that seen in IFA stained images (Supplementary Fig. S3).

### Automated TGL microscopy

In order to make the TGiA assay useful for potential clinical application, it is necessary to automate the microscopic imaging. We have previously reported a new development in orthogonal scanning automated microscopy (OSAM)^36^ integrated with time-gated modality for the detection of long-lived luminescent targets.

Supplementary Fig. S4A shows the circular outline of the membrane filter (13 mm diameter) that captured the cells. The area that OSAM performs the scanning is shown as a square shape with an area of 15 by 15 mm. Scanning of this square area was completed in 3.3 min at a spatial resolution of 1.7 μm. The OSAM scanning output created a 2D-map on the computer screen using in-house developed LabVIEW software that determines where on each slide the stained PCa cells (bright dots) were located as shown in Supplementary Fig. S4B. The position of each identified PCa cell (bright dots) is stored in software (LabVIEW) that also can be precisely re-viewed under the bright field, TGL mode and the DAPI channels. This provides the opportunity to visualize and re-examine the detected targets through the eyepiece of the microscope if further confirmation of the detected cells is needed. Some of the bright dots are multiple target cells that aggregated together but the single cells comprising the aggregate were counted separately. Therefore, OSAM microscopy is capable of the detection of low number of spiked and labelled PCa cells (10, 50 and 100 DU145 cells spiked into urine 1 mL-Figure S4B-A, B-B and B-C) and we have shown that OSAM has the instrumental capability of single luminescent-labelled cell detection (Supplementary Figure S4B-D).

### Raman-active immunolabeling assay (RiA)

The second, non-microscopy based, technique uses a Raman-active immunolabeling assay (RiA) to avoid the problems of auto-fluorescence by choosing a Raman-active nanotag excited in the near infrared (785 nm). The RiA is illustrated schematically in Supplementary Fig. S5. The typically low-intensity of Raman Stokes spectrum of the reporter molecule on the nanotag is greatly increased using surface-enhanced Raman scattering (SERS), wherein the Raman scattering signal of the reporter molecule is enhanced typically a million-fold by the large optical fields generated by way of plasmonic effects at the nanoscale surfaces of noble metal nanoparticles (gold nanoparticles, named SERS nanotags). ^37,38^SERS nanotags can be conjugated to a specific antibody to target cells, resulting in a Raman signal which is proportional to the number of target cells in the observation aperture of a handheld Raman spectrometer. For the present demonstration of this method, we have used PCa specific MIL-38 antibody conjugated SERS nanotags. Supplementary Fig. S6 shows the scheme of preparation of SERS nanotags and the characterization of SERS nanotags by Raman, UV–Vis absoprtion spectra and TEM images were shown in Fig. S7. For clinical application, it is of considerable practical importance that the Raman spectrometer used to detect and quantify the Raman emission is a handheld device rather than a laboratory benchtop device. ^39,40^The RiA assay specficity (Fig. S8) has been tested, and the limit of detection (LOD) of RiA was determined as 4 PCa cells/mL in PBS (Fig. S9) and 10 cells/mL in urine samples (Fig. S10).

### Application of TGiA and RiA assays for detection and quantification of PCa cells in patient urine samples

Analysis of freshly collected urine specimens from PCa patients was performed in triplicate by both TGiA and RiA assays compared with control urine samples from healthy male volunteers The number of PCa cells detected in three replicate experiments with TGiA and RiA for each patient is shown in Table 1.

Patient 1 was clinically evaluated as high-risk prostate cancer due to the increasing PSA level (from 8.1 to 13 ng/mL in 4 months) and magnetic resonance imaging (MRI) evidence of PCa. The biopsy was not performed by the time of this study, thus Gleason score was not available. The TGiA assay revealed the presence of 14, 8 and 11 PCa cells in the urine sample in three replicate analysis. The RiA analysis showed a distinctive specific Raman peak of around 10 positive cells per mL in each of the replicates.

Patient 2 was diagnosed with high-risk prostate cancer due to high Gleason score (9 (4 + 5)) and skeletal metastasis to T9 vertebra. This patient had a moderately elevated PSA level (7.9 ng/mL). The TGiA assay identified 3, 11 and zero PCa cells in the replicate urine samples in which the RiA assay gave a positive signal corresponding to less than 10 cells per mL, 10 cells per mL and no signal in the three replicates.

Patient 3 had intermediate-risk prostate cancer with recommendation for radical prostatectomy with Gleason score of seven and rapid increase of PSA level from 6.3 to 8.7 ng/mL within a month. The TGiA analysis revealed the presence of 20, 12 and 7 PCa calls in replicate samples. The RiA analysis detected above 10 PCa cells per mL in the first replicate and around 10 PCa cells per mL in the other two replicates.

Patient 4 had a localised tumour in his prostate with Gleason score seven and high PSA level (14 ng/mL). This patient was classified as low to intermediate-risk prostate cancer based on the size of the tumour and no progression of cancer for several years. Active surveillance was advised as his cancer was not aggressive. No PCa cells were identified in the sample replicates either by TGiA or RiA assays.

Patient 5 had partial transurethral resection of prostate in January 2017. Histological analysis of the removed tissue identified a small isolated nodule of PCa with Gleason score of nine. Since the biopsy from the remaining prostate tissue was negative and the PSA level remained very low (0.24 ng/mL), the patient was classified as low risk. Since then, he was on 6-monthly surveillance with PSA being very low at 0.15–0.3 and MRI in 2017, 2018 and 2021 which showed no lesions. Overall, there has been no evidence of residual disease or progression, The diagnosis of significant prostate cancer in this case was thus based on radiological, clinical and bio marker results and Patient 5 is therefore pT1a N0M0 but high risk Grade group 5 cancer. The analysis of their urine by both TGiA and RiA assay was negative.

Note that the volume of urine in each patient’s assay was initially adjusted based on the total urinary cell count; within the range of 100–200 cells. This adjustment was performed based on the initial optimization of labelling and imaging of spiked PCa cells (DU145) into control urine for the TGiA assay (Supplementary Information, Fig. S1) and the same adjustment was performed for the RiA assay. As shown in Table 1, patients 1, 2 and 5 had total cell counts within the required range with one mL of urine. However, for patients 3 and 4, the volume of urine sample needed to be adjusted to 3 mL and 2 mL, respectively. Accordingly, the mean of the PCa cells detected by each assay was normalized for 1 mL of urine in order to provide an accurate comparison of the clinical data of patients with the detected PCa cells (Table 1). Parallel control experiments were performed using urine samples of 5 healthy male volunteers with a zero result in both TGiA and RiA assays.

**Figure 2:**
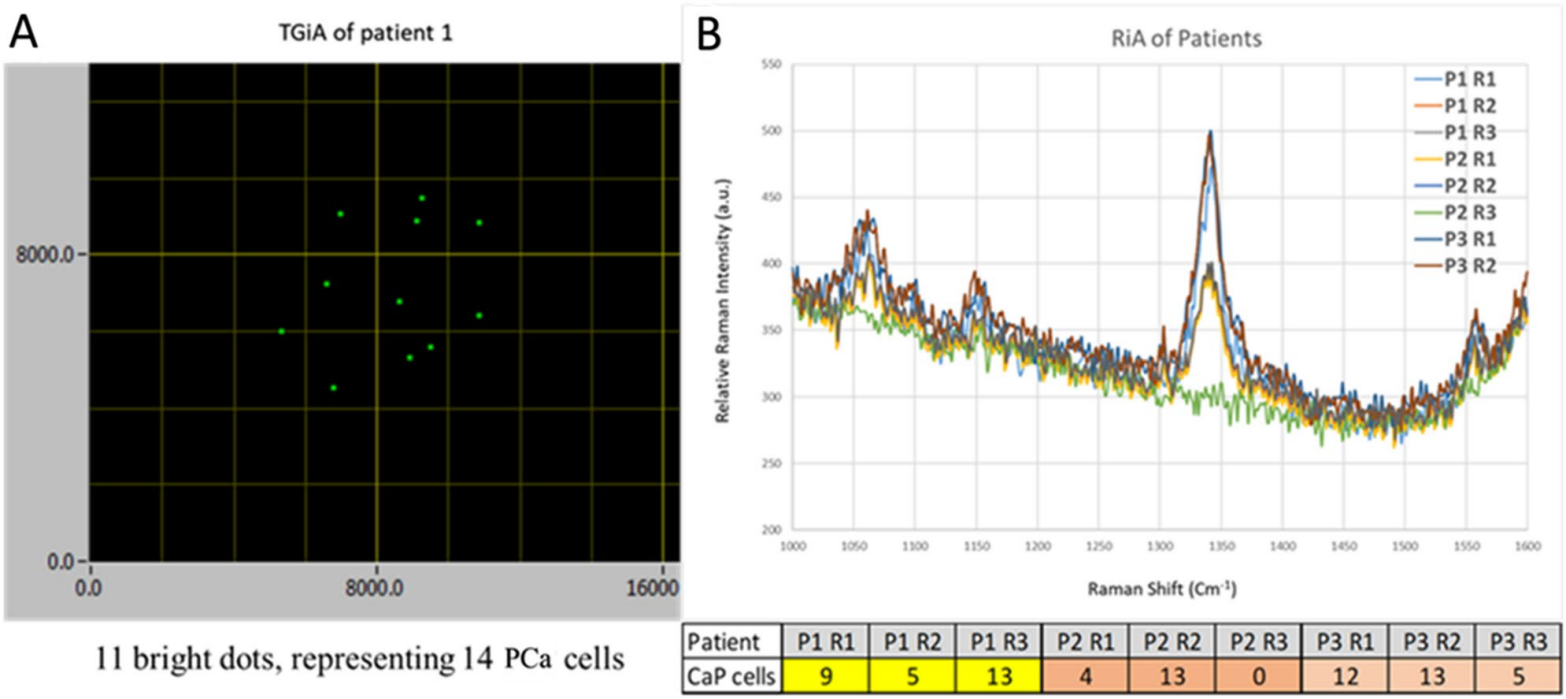
(**A**) Representative image of PCa cells in TGiA OSAM map of patient 1 shows the screenshot of 2D-map of the OSAM scanning output of the TGiA analysis (11 bright dots are representing 14 PCa cells as some PCa cells aggregated together, and (**B**) Raman spectra of 3 replicates (R1, R2, R3) of RiA assay for patient 1, 2 and 3 (P1,P2,P3).

The number of cells in urine of patients 1, 2 and 3 was found to be in the range of 4–13 PCa cells per mL in the RiA assay that is correlated with the numbers of 3–20 PCa cells detected by TGiA (Table 1). Representative data from both assays of patient urine are shown in Fig. 2.

**Figure 3:**
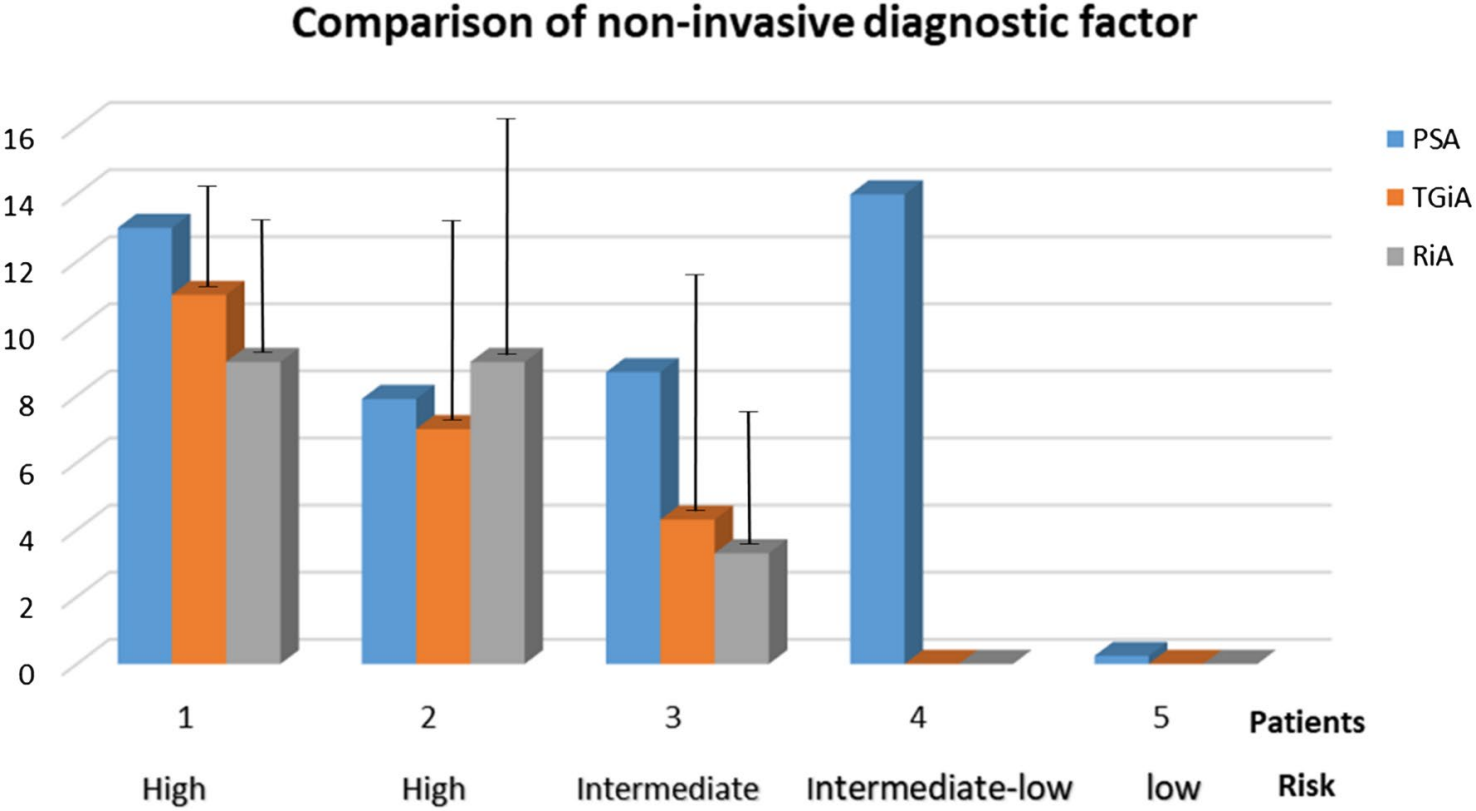
The reported PSA level (pathology result) and the numbers of PCa cells detected by TGiA and RiA assays (three replicates) in a small cohort of patients’ urine with different clinically determined risk.

Figure 3 plots the normalized mean of the number of PCa cells (detected by both assays) and the PSA level and clinically determined risk, for each patient. For all patients, the detected numbers of PCa cells in both assays are strongly correlated with the clinical risk assessment. Patients 1 and 2 with a higher risk assessment, have high PSA and PCa cells detected by both assays. Patient 3 has intermediate risk and shows lower PSA and detected PCa cells by both assays. Patients 4 and 5 show low risk on clinical assessment but show highly disparate PSA levels. Interestingly, no PCa cells were detected in the urine using either assay. In particular, in regard to correlation with PSA, Patient 4 had a very high PSA level but was assessed from clinical history as having medium/low risk and had zero PCa cell count by both assays. Despite the very limited numbers of tested urine samples of PCa patients in this study, the data presented in this proof-of-principle work has demonstrated that whole-cell PCa counts in the range 3–20 cells/mL of urine are enabled by both TGiA and RiA assays and are highly correlated with the clinically determined risk of PCa patients (Fig. 3).

### Advantages of TGiA and RiA assays over current urine cytology assays

As described, both assays provide a very high level of detection sensitivity (in the range 3–20 cells/ml of urine). In comparison, the conventional urine cytology tests such as IFA or colorimetric microscopic assays for the detection of urinary PCa cells can only achieve a minimum sensitivity of about 10^3^ cells/mL of the original urine sample^22,23^ as the traditional cytospin approach requires at least one thousand cells for consistent cell capture from urine, which is often not present in PCa urine specimens. Capturing low numbers of PCa cells from urine using the described filter membrane adapted from the method of Nickens et al.^32^. is an important aspect of this work in increasing the applicability of the TiGA cytology assay whereas the RiA does not require cell capture. Eskra et al. have concluded that while cytology techniques can deliver high specificity, the low sensitivity represents a severe limitation to the practicability of the current approaches^21^. The two new urine cytology assays described in the manuscript offer the specificity of the MIL 38 antibody for the Glypican antigen expressed in PCa cells with the increased sensitivity derived from the improved cell detection technologies described.

## Conclusion

In summary, we have demonstrated two state-of-art molecular cytology approaches which allow limits of detection of a few prostate cancer cells per millilitre of human urine.

The correlation of urinary prostate cancer cell counts with clinically determined risk indicates that these two described molecular cytology tests offer great promise for application as non-invasive diagnostics of active prostate cancer. The TGiA filtration test uses a combination of europium-2Ab-MIL-38-probe for staining the GPC-1 cancer antigen on urinary PCa cells and uses automated time-gated microscopy to eliminate the auto-fluorescence background to provide significant improvement in detection sensitivity over existing urinary cytology methods. This technology gives single cell detection sensitivity with the lowest detectable cell numbers of 3 PCa cells/mL urine (~ 8 h assay) and eliminates the need for the expert, time-consuming process of the standard cytoslide analysis by epifluorescence microscopy. On the other hand, we have demonstrated that the RiA test using the MIL-38 antibody has a LOD of 4 PCa cells/mL of urine sample using a handheld Raman spectrometer, with the time required for the assay, including sample preparation and measurement, of only 45 min.

We recognise that the small number of PCa patients is a limitation of this study, but the ability of sensitive and specific detection of PCa cells in patient urine, demonstrated by both assays, shows the exciting potential for new rapid and sensitive non-invasive prostate cancer diagnosis. These detection platforms may also be applicable for other analyses where cell based surface biomarkers are of diagnostic and/or prognostic value.

### Supporting information

For cell lines preparation, fixation and filtration of spiked DU145 cells and urine samples preparation of europium chelate conjugated to secondary antibody, specificity, and sensitivity and automated microscopy of TGiA assay. Preparation of RIA nanotags conjugated to primary antibody, Raman-active Immunolabeling Assay (RiA) specificity and sensitivity and LOD of the RiA assay are also described in SI. The data generated during this study are available in the Supporting Information (SI), further details can be provided by corresponding authors upon request.

## Supplementary Information

Supplementary Information.
