# Supplementary material for: Detection of rare prostate cancer cells in human urine offers prospect of non-invasive diagnosis
Source: Sci Rep. 2022 Nov 2;12:18452. doi: 10.1038/s41598-022-21656-9 (PMC9630382; doi:10.1038/s41598-022-21656-9)
Supplement: Supplementary file 1 — Supplementary Information. [file 41598_2022_21656_MOESM1_ESM.docx]

**Supplementary information:**

Detection of Rare Prostate Cancer Cells in Human Urine Offers Prospect of Non-Invasive Diagnosis

Nima Sayyadi^1,2^, Irene Justiniano^1,3^, Yan Wang^2,4^, Xianlin Zheng^2,4^, Wei Zhang^1^, Lianmei Jiang^1,2^, Dmitry M. Polikarpov^2,5^, Robert D. Willows^1^, David Gillatt^5^, Douglas Campbell^3^, Bradley J. Walsh^3^, Jingli Yuan^6^, Yiqing Lu^2,7^, Nicolle H. Packer*^1,2^, Yuling Wang*^1,2^ and James A. Piper^2,4^

^1^ School of Natural Sciences, Macquarie University, Sydney, Australia.

^2^ ARC Centre of Excellence for Nanoscale Biophotonics (CNBP), Macquarie University, Sydney.

^3^ Minomic International Ltd, Macquarie Park, Sydney, Australia.

^4^ Department of Physics and Astronomy, Macquarie University, Sydney, Australia.

^5^ Faculty of Medicine and Health Sciences, Macquarie University, Sydney, Australia.

^6^ State Key Laboratory of Fine Chemicals, School of Chemistry, Dalian University of Technology, Dalian, China.

^7^ School of Engineering, Macquarie University, Sydney, Australia.

* Corresponding authors: [nicki.packer@mq.edu.au](mailto:nicki.packer@mq.edu.au), [yuling.wang@mq.edu.au](mailto:yuling.wang@mq.edu.au)

The Supplementary Information contains the data on PCa cell line cells (DU145) spiked into urine of healthy male volunteers for the development and optimization of the TGiA and RiA assays before carrying out the tests in PCa patients’ urine as presented in the main text of the manuscript.

**Materials and Methods**

**Cell lines:** The prostate cancer cell line (DU145, ATCC HTB-81) and bladder cancer cell line (C3) were maintained in RPMI 1640 medium supplemented with fetal bovine serum (FBS) (10% w/v) with 1 mM glutamine at 37 °C in a humidified 5% CO_2_ atmosphere. Cells were grown to 80% confluence and then washed three times in PBS to remove excess medium. Cell monolayers were detached from the culture flask by adding EDTA (2 mM) in PBS and incubating at 37 °C for 15 min. The cells were then collected by centrifugation at 300g for 5 min. resuspended in PBS and counted with a TC20TM Automated Cell Counter (Bio-Rad) by analysing 10 μL of cells mixed with 0.4% w/v Trypan blue (1:1, v/v).

**Cell capture efficiency by filtration of spiked DU145 cells for TGiA detection:**

The cell capture efficiency was tested initially by spiking of 10, 50, or 100 cells DU145 cultured cells, counted by automated cell counter (Bio-Rad), into 1 mL of PBS or 1 mL of pre-cleaned urine (supernatant of urine centrifuged at 3,000 g for 15 min). The cells were then fixed (Saccomanno fixative, 1:1 (v/v) ratio, incubation at RT for 2 hr) and filtered via disposable syringe (1 to 5 ml) in a filtration assembly system using a polycarbonate hydrophilic membrane filter (8µm pore size) in a pop-top filter membrane holder (Figure S1, A). The filter membrane was first pre-wet by passing 5 ml of PBS before it was used to filter the cells.

Capture efficiency of spiked DU145 cells was determined by manual counting of the DAPI stained filtered cells using an epifluorescence microscope. Captured cells were initially quantified and visualized by addition of 1 mL DAPI solution (DAPI 5 μL [2 μg/mL] in 1 mL PBS) to the filter assembly system via syringe and incubation for 5 min. The filter was then washed 3 times with PBS (1 mL) using a disposable syringe. Finally, the filter was removed from the filter assembly and mounted on a microscope slide. Mounting media (20 μL PBS with 70% v/v glycerol) was added and a coverslip put on top of the filter. The cells captured were counted (DAPI nuclei staining) visually under Olympus BX51 upright fluorescence microscope.


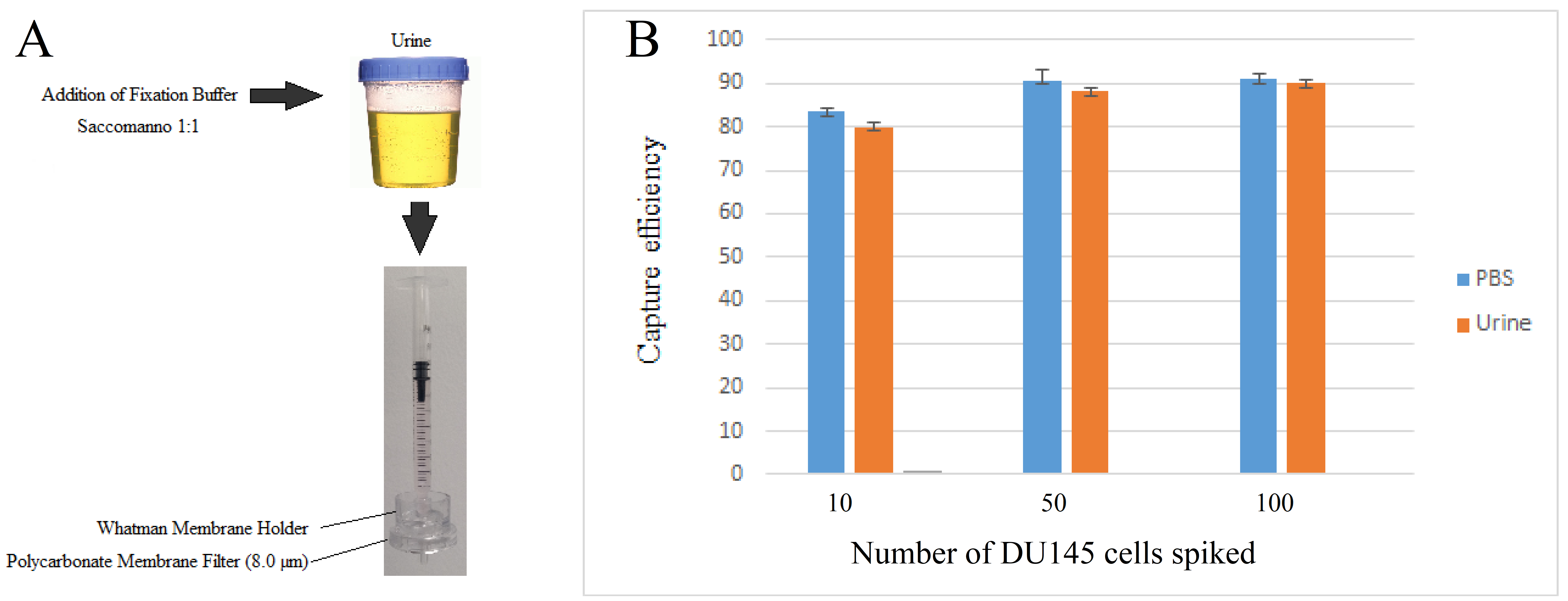


**Figure S1**: (A) A schematic of the PCa cells filtration and efficient capture of urinary cells (B) Average capture efficiency of PCa cells (DU145 cells) in PBS and pre-cleaned urine, visualised after DAPI staining using conventional microscope. (n=3)

Time-gated luminescence imaging was performed using orthogonal scanning automated microscopy (OSAM) on TGL mode and images were captured using a DP72 colour camera set for ASA speed of 200 and exposure period of 4.0 s. All images captured were stored as TIFF files and analysed without modification. DAPI stained images were captured using the same UV lamp and DAPI filter set up at a 4 ms exposure time.

A conventional epifluorescence microscope (Olympus BX51 upright fluorescence microscope) was used to evaluate the fluorescently labelled cells (secondary antibody-Alexa Fluor 488) under bright-field, DAPI and FITC channel. FITC fluorescence and DAPI imaging were carried out with a 100 W mercury arc lamp and a DAPI and FITC filter set to 10 ms and 300 ms exposure times respectively.

The SNR was determined by dividing the average of mean signal intensity of target cells by the average of mean signal intensity of background non-target cells (N) as detailed in our previous study.^1-3^ Briefly, labelled DU145 cells with TGiA or IFA were randomly selected and the mean signal intensities of the whole target cell areas were quantified using the ImageJ histogram mode (red channel used for TGiA and green channel for IFA). The mean signal intensity of single selected cells from images of 10 microscope slides was then averaged to determine the target signal (S). The noise (N) was quantified in the same manner on background non-target cells areas.

**Specificity and sensitivity of the TGiA microscopic assay**

Eu-Probe TGiA staining of spiked DU145 cells into control urine (50 cells per 1 mL urine) showed that the Eu-Probe selectively labelled the DU145 cells compared to the urinary cells. In the bright field of the microscope (representative image, Figure S2A) the epithelial cells can be easily distinguished from DU145 prostate cells due to the larger size of epithelial cells and smaller nucleus compared to the PCa cells. Note the small bright circles and dark cylindrical shapes in the bright field are due to the membrane filter matrix. The DAPI channel shows the nucleus in both DU145 and epithelial cells in the field of view (Figure S2B). TGL imaging shows only DU145 cells were selectively stained by the Eu-Probe (red colour) from the luminescence emission of europium (III) at 615 nm (Figure S2C).

**
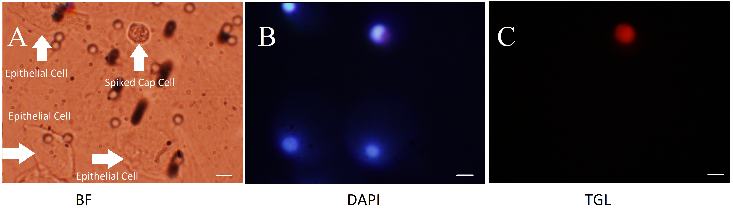
Figure S2:** Immunoluminescence staining of DU145 PCa cultured cells spiked into control urine. Eu-Probe selectively labelled spiked DU145 cells (A) bright field, arrows pointing to epithelial cells and PCa (DU145) cells [small bright circles and dark cylindrical shapes are due to the membrane filter matrix] (B) DAPI (C) TGiA shows selective staining of the DU145 cell. The scale bar represents 10 μm.

To demonstrate the higher detection sensitivity of TGiA compared to IFA in staining of DU145 cells, we compared the Eu-Probe with the Alexa-Fluor 488 probe for staining of spiked DU145 cells filtered from urine (50 cells spiked in 1 mL urine). As shown in Figure S3A, DU145 cells were stained background free by the Eu-Probe as imaged by a TGL microscopy system. On the other hand, the Alexa-Fluor 488 probe also stained the DU145 cells, but the green 488 nm fluorescent emission overlapped with the auto-fluorescence background of other cells filtered from the urine matrix (Figure S3B).

The detection sensitivity or signal-to-noise ratio (SNR) of TGiA compared to IFA imaging of labelled DU145 cells was determined. As shown in Figure S3C, the average of mean signal intensity (S) in TGiA imaging was 1.7 times higher than with IFA, while the average intensity of noise (N) in TGiA images was nearly 6 fold lower than in IFA images. Consequently, the average SNR achieved for TGiA staining was about 10 times higher than that seen in the IFA stained images. Eliminating the auto-fluorescence background of urine specimens is a key advantage of TGiA microscopic detection of PCa cells filtered from urine.


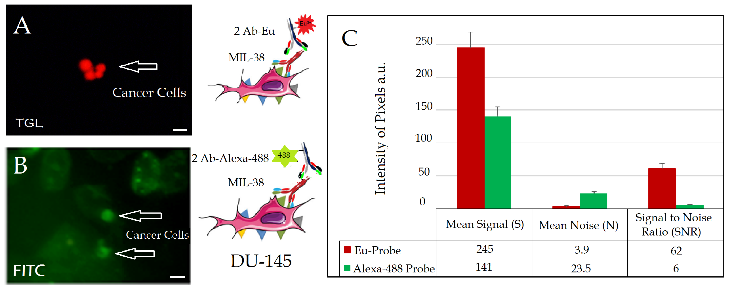
**Figure S3:** Staining of PCa cells (DU145) spiked into the control urine (50 cells per 1 mL urine) containing epithelial cells is shown with (A) Eu-Probe staining with TGL imaging (B) MIL38-Alexa-Fluor 488-Probe staining using a FITC channel with an epifluorescence microscope, The scale bar represents 20 µm. C) Average of mean signal intensity of the target DU145 cells (S), average of mean signal intensity of non-target cells, noise (N) and signal to noise ratio (SNR). Standard deviation (SD) is shown (n = 3).

**Single cell detection by TGiA**

To investigate the limits of detection of PCa cell imaging by OSAM, cells (10, 50 and 100 DU145 cells) were spiked into urine (1 mL), labelled with Eu-Probe and then imaged by OSAM. As shown in the Figure S4 (A, B & C), the number of detected cells correlated well with the number of spiked cells (17, 47 and 98 cells were detected and counted respectively) as shown in the 2D-map from the computer screen. Note some of the green dots are multiple target cells that aggregated together but the single cells comprising the aggregate were counted separately to determine the cell recovery. To validate the data, the detected cells (green spots on 2D map) were visualized through the eyepiece of microscope under bright field, DAPI and time-gated modality.

Finally, to test if a single luminescent-labelled cell can be detected by OSAM, a single luminescent-labelled cell was transferred three times to the filter and analysed and each time the single cell was detected (Figure S4D). Note: the single labelled cell was manually moved to the filter and used to examine the capability of OSAM in single cell detection so it does not imply that a spiked single cell was labelled and detected.


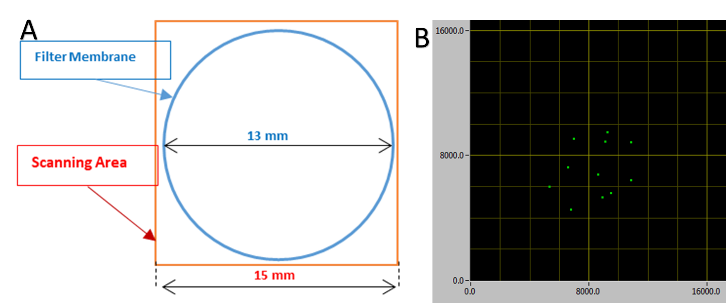


**B.**

*
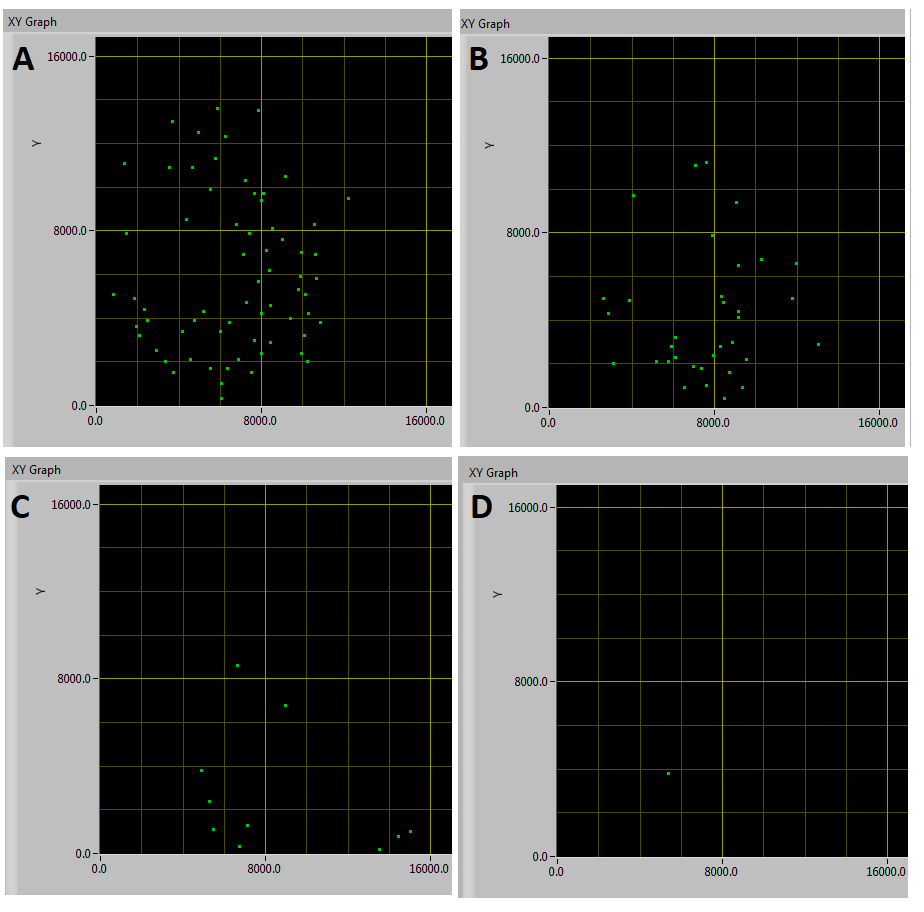
*

Single PCa Cell

**Figure S4**: (A) Scheme representing filter membrane (circular shape) and the scanning area (square shape). (B) 2D-map of OSAM output of TGiA labelled (B-A) 100 (B-B) 50 and (B-C) 10 DU145 cells spiked into the urine (1 mL) samples. Around 98 labelled cells in A, 47 cells in B and 17 cells in C were detected as seen in green. The green spots are single or multiple aggregated cells. In 2D-map (B-D) a single TGiA labelled DU145 cell was manually transferred to the slide and detected by OSAM.

**Working principle of RiA method**:


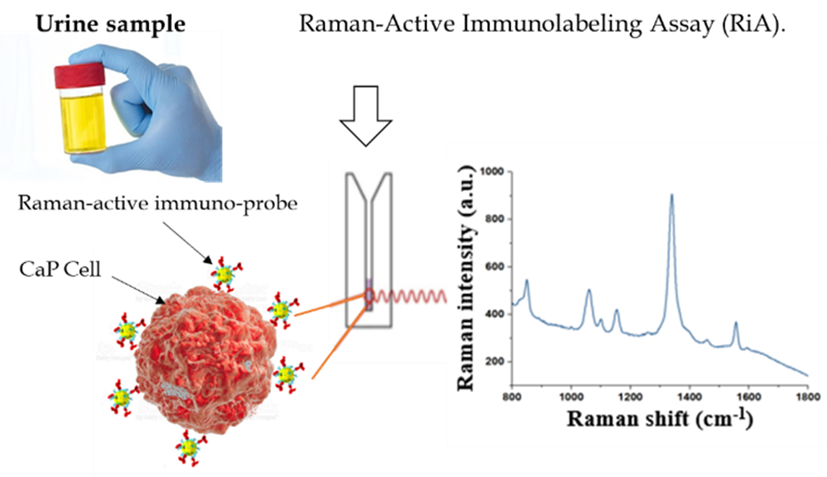


**Figure S5;** Schematic of the Raman-Active Immunolabeling Assay (RiA).

RiA was used for detection of PCa cultured cells (DU145) spiked into urine samples is illustrated in Figure S5. RiA consisted of AuNPs (50 nm) and a Raman reporter molecule (DTNB, with distinct peak at 1337 cm^-1^) and antibody (MIL-38) as described above. The SERS peak at 1337 cm^-1^ demonstrates the binding of Raman reporter molecules (DTNB) onto AuNPs and was consistent after conjugation to antibody (SERS-DTNB-MIL38) (Figure S6A). Successful conjugation of AuNPs with antibody (MIL-38) was further confirmed by UV-visible absorption analysis of AuNPs with and without the antibody MIL-38. UV-Vis spectrum of the antibody-conjugated AuNPs shows that the surface plasmon band of AuNPs shifted slightly from 536 to 543 nm with a distinct peak absorption of antibody at 280 nm (Figure S6B). A typical transmission electron microscopy (TEM) image of the SERS nanotags showed a thin protein layer coated onto AuNPs surface after conjugation to the antibody as shown in Figures S6C and D.


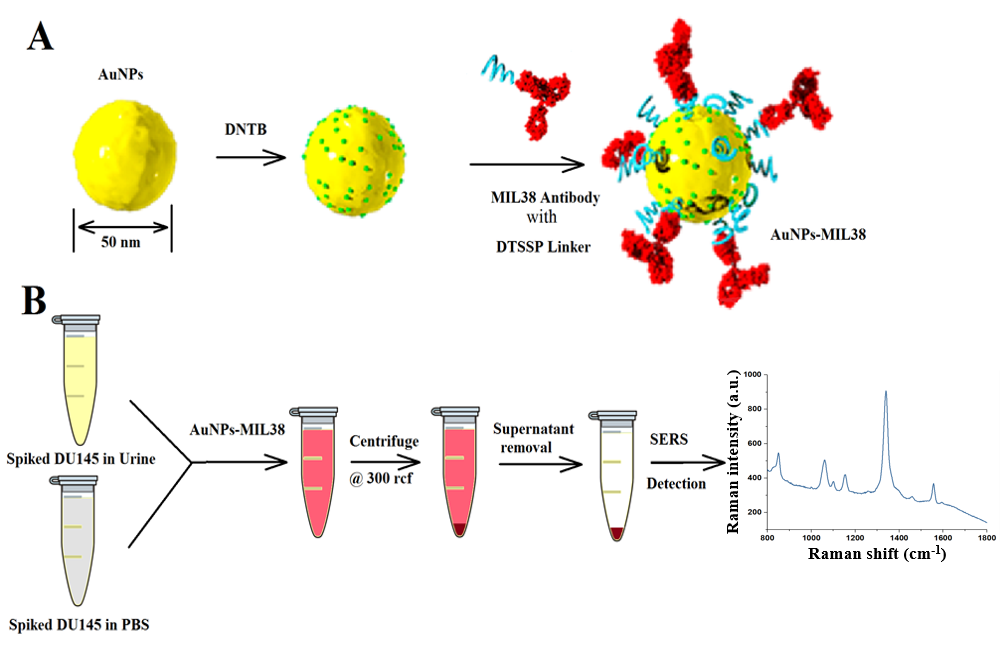


**Figure S6**: (A) The process for fabricating AuNPs with DTNB Raman reporter and conjugation to antibody (MIL-38) attached to a DTSSP linker. This conjugation provided the final product AuNPs-DTNB-DTSSP-MIL-38, which is termed “SERS nanotags” (B) A scheme showing the workflow for incubating the spiked DU145 cells in urine and patients’ urine samples with SERS nanotags (colloidal suspension) with detection by a Raman spectrometer.


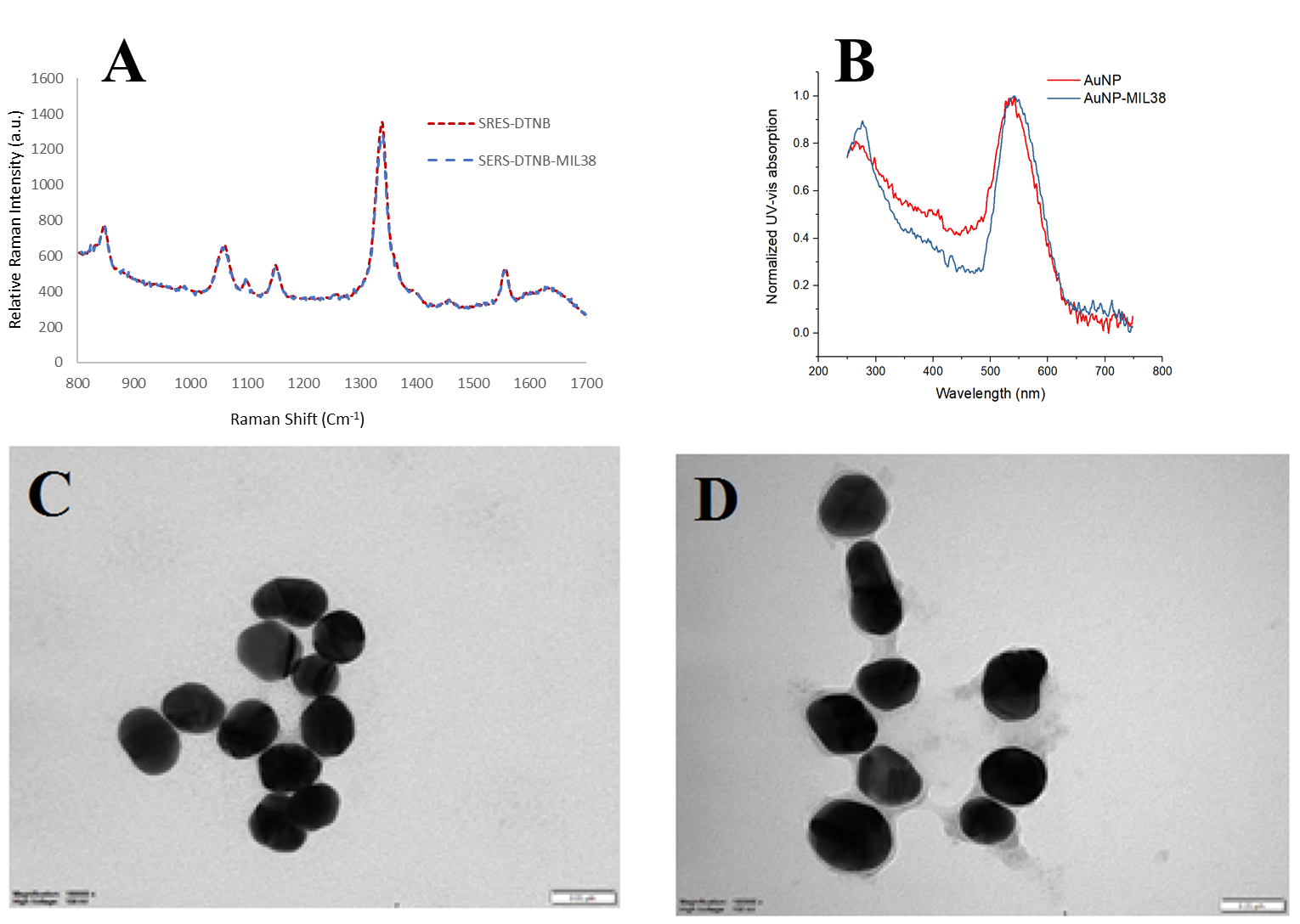


**Figure S7**: (A) A Raman spectrum of SERS-DTNB and SERS-DTNB-MIL38 (distinct peak at 1337 cm^−1^). (B) UV−vis spectra of AuNPs and AuNPs-MIL-38. (C) A TEM image of the AuNPs and the (D) AuNPs-MIL-38.

**Specificity and sensitivity of the RiA assay*:*** To test the RiA assay specificity, we used two cultured cell lines [DU145 (PCa positive) and C3 (negative control)]. Initially, SERS nanotags with and without conjugation to antibody (MIL-38) were incubated with the DU145 cells (1000 cells/mL). The data collected revealed that SERS nanotags without antibody did not bind to the cells and the cells did not display a Raman signal after washing (Figure S7A, A). In contrast, a high signal was detected when the MIL-38 antibody was conjugated to the SERS nanotags (Figures S7A, B).

The detection of DU145 cells spiked into urine (10,000 cells/mL urine), was compared with the same amount of spiked non-target bladder C3 cells. As shown in Figure S7A (C and D) the intensities of the Raman signals for the DU145 and C3 cells were observed to be significantly different; the relative Raman intensity of DU145 cells (Figures S7A, C) was at least ~10 times higher compared to that of C3 cells (Figures S7A, D). When the C3 cells were tested at a lower cell number (<1000 cells/mL), a Raman peak was not visible, and this confirmed the high specificity of the SERS nanotags assay for cell surface marker detection, with minimum to no non-specific noise signal.


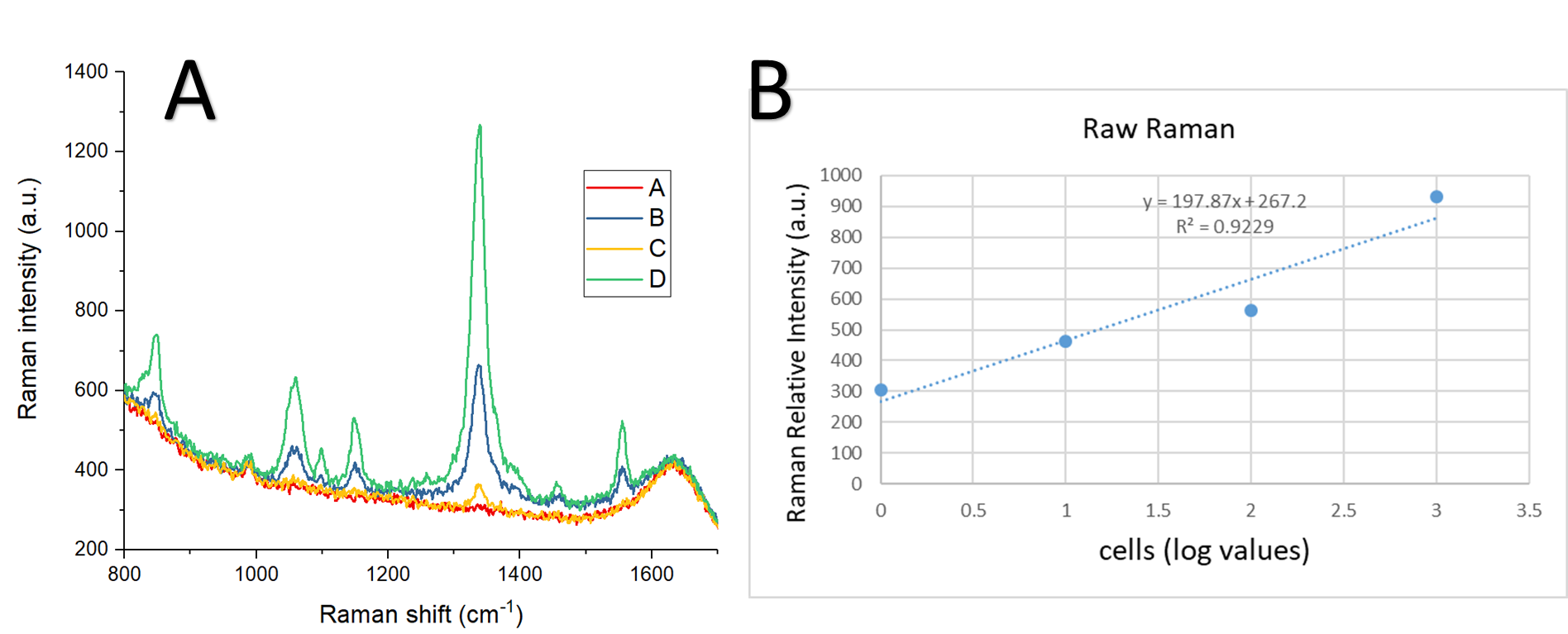


**Figure S8:** (A) Raman spectra of MIL-38 binding to DU145 cells spiked into urine: A) SERS nanotags without MIL-38, B) SERS-MIL-38 nanotags against DU145 cells (1000 cells/mL), C) SERS-MIL-38 nanotags against C3 cells (GPC1-negative) (10,000 cells/mL) D) SERS-MIL-38 nanotags against DU145(10,000 cells/mL). (B) The Raman intensity of the SERS nanotags following titration of the DU145 cells (1000, 100, 10 and 0) cell per 1 mL of control urine and limit of detection (LOD) was quantitated according to the linear equation obtained from the spiked cells in urine of 10 cells in 1 mL of control urine.

**Limit of detection (LOD) of the RiA assay:** To investigate the detection sensitivity of the RiA assay, we spiked different numbers of DU145 cultured cells (1000, 100, 10, and 0) per 1 ml of urine and incubated with SERS nanotags as indicated in Figure S7B. The average Raman peak intensity of three replicates (at 1337 cm^-1^) was plotted versus the log number of DU145 cells spiked into urine sample. As shown in Figure S7B, Raman intensities showed a positive correlation with increasing cell numbers, and limit of detection was quantitated according to the linear equation obtained from the spiked cells in urine Figure S7B (y=197.87x+267.2, R^2^=0.923). Accordingly, a limit of detection (LOD) of 4 cells/mL in PBS and 10 cells/mL in urine were determined according to the three times of signal-to-noise of blank (Figure S8 and S9)


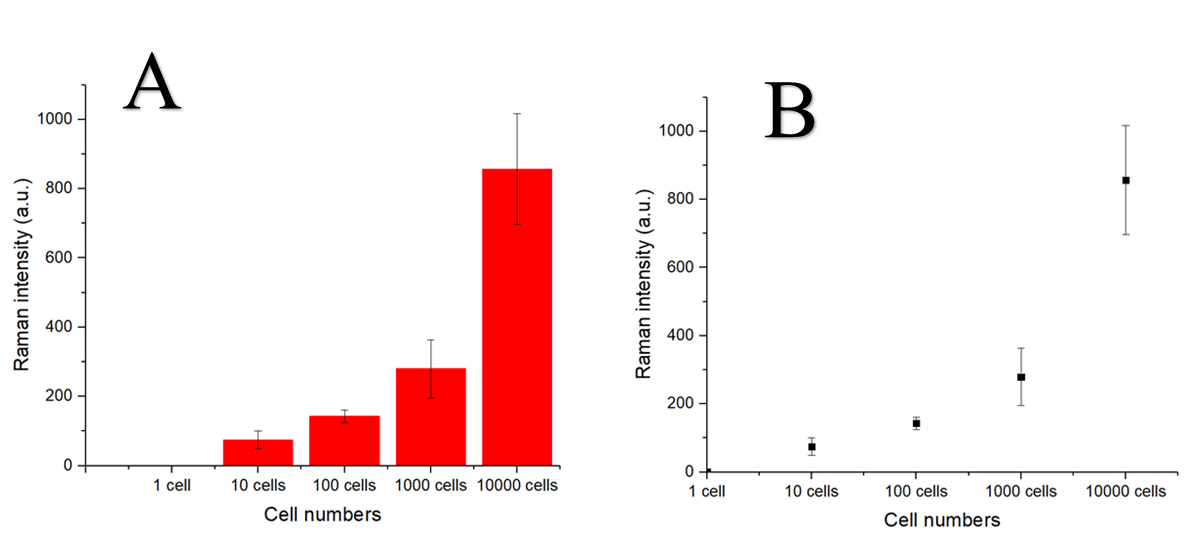


**Figure S9:** (A) The Raman intensity of the SERS Nano-tag of titration of the DU145 cells (10,000, 1000, 100, 10 and 1) cell per 1 mL PBS (B) limit of detection (LOD) of 4 cells 1 mL in PBS.

**
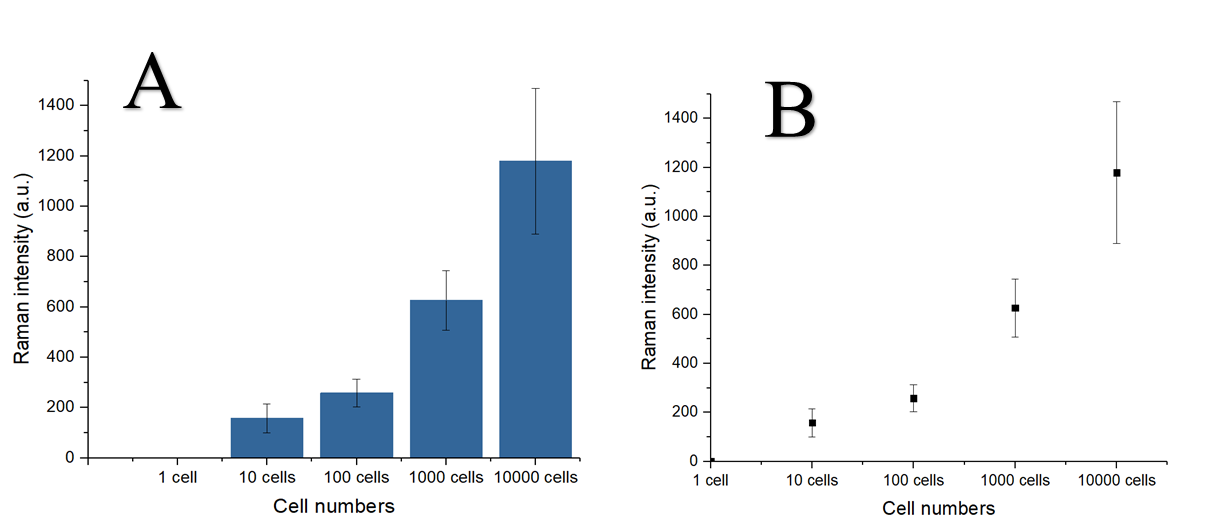
**

**Figure S10:** (A) The Raman intensity of the SERS Nano-tag of titration of the DU-145 cells (10,000, 1000, 100, 10 and 1) cell per 10 mL in control urine. (B) Limit of detection (LOD) of 10 cells in 10 mL of control urine.

**References:**

1 Sayyadi, N., Connally, R. E. & Try, A. A novel biocompatible europium ligand for sensitive time-gated immunodetection. *Chemical Communications* **52**, 1154-1157 (2016).

2 Sayyadi, N. *et al.* A Novel Universal Detection Agent for Time-Gated Luminescence Bioimaging. *Scientific reports* **6** (2016).

3 Sayyadi, N. *et al.* Time-Gated Luminescent In Situ Hybridization (LISH): Highly Sensitive Detection of Pathogenic Staphylococcus aureus. *Molecules* **24**, 2083 (2019).
